# Comparison of medical history documentation efficiency and quality based on GPT-4o: a study on the comparison between residents and artificial intelligence

**DOI:** 10.3389/fmed.2025.1545730

**Published:** 2025-05-14

**Authors:** Xiaojing Lu, Xinqi Gao, Xinyi Wang, Zhenye Gong, Jie Cheng, Weiguo Hu, Shaun Wu, Rong Wang, Xiaoyang Li

**Affiliations:** ^1^Department of Medical Education, Ruijin Hospital Affiliated to Shanghai Jiao Tong University School of Medicine, Shanghai, China; ^2^WORK Medical Technology Group LTD, Hangzhou, China; ^3^Shanghai Resident Sandardized Training Center, Shanghai, China

**Keywords:** artificial intelligence, GPT-4o, medical history documentation, quality, efficiency

## Abstract

**Background:**

As medical technology advances, physicians' responsibilities in clinical practice continue to increase, with medical history documentation becoming an essential component. Artificial Intelligence (AI) technologies, particularly advances in Natural Language Processing (NLP), have introduced new possibilities for medical documentation. This study aims to evaluate the efficiency and quality of medical history documentation by ChatGPT-4o compared to resident physicians and explore the potential applications of AI in clinical documentation.

**Methods:**

Using a non-inferiority design, this study compared the documentation time and quality scores between 5 resident physicians from the hematology department (with an average of 2.4 years of clinical experience) and ChatGPT-4o based on identical case materials. Medical history quality was evaluated by two attending physicians with over 10 years of clinical experience using ten case content criteria. Data were analyzed using paired *t-*tests and Wilcoxon signed-rank tests, with Kappa coefficients used to assess scoring consistency. Detailed scoring criteria included completeness (coverage of history elements), accuracy (correctness of information), logic (organization and coherence of content), and professionalism (appropriate use of medical terminology and format), each rated on a 10-point scale.

**Results:**

In terms of medical history quality, ChatGPT-4o achieved an average score of 88.9, while resident physicians scored 89.6, with no statistically significant difference between the two (*p* = 0.25). The Kappa coefficient between the two evaluators was 0.82, indicating good consistency in scoring. Non-inferiority testing showed that ChatGPT-4o's quality scores fell within the preset non-inferiority margin (5 points), indicating that its documentation quality was not inferior to that of resident physicians. ChatGPT-4o's average documentation time was 40.1 s, significantly shorter than the resident physicians' average of 14.9 min (*p* < 0.001).

**Conclusion:**

While maintaining quality comparable to resident physicians, ChatGPT-4o significantly reduced the time required for medical history documentation. Despite these positive results, practical considerations such as data preprocessing, data security, and privacy protection must be addressed in real-world applications. Future research should further explore ChatGPT-4o's capabilities in handling complex cases and its applicability across different clinical settings.

## Introduction

With the continuous advancement of medical technology, physicians are shouldering increasingly greater responsibilities in clinical practice (1). The collection and documentation of medical history has become an indispensable part of daily work, particularly in the management of hospitalized patients. Medical history serves not only as a crucial basis for diagnosis and treatment but also as a key document for legal and insurance purposes (2). Therefore, accurate and comprehensive documentation is vital for patient outcomes and the quality of healthcare services (3).

However, in busy hospital environments, resident physicians often face tremendous time pressure (4). Particularly in China, they are required to complete high-quality medical history documentation within limited time frames, which undoubtedly presents a significant challenge. This situation may affect the quality of documentation, leading to reduced work efficiency and increased professional burnout among physicians.

In recent years, the application of Artificial Intelligence (AI) technology in healthcare has been expanding, bringing new possibilities for improving the quality and efficiency of healthcare delivery (5–7). Among these technologies, Natural Language Processing (NLP) has demonstrated remarkable potential in medical text generation and analysis (8). The emergence of large language models like GPT-4o, in particular, has made AI-assisted medical documentation possible, potentially transforming traditional documentation methods (9).

GPT-4o (10), through its analysis of vast amounts of language data, can generate structured and coherent text, establishing a solid foundation for its application in medical documentation (11). However, despite AI's promising prospects in healthcare, its effectiveness and reliability in actual clinical settings still require further validation (12). Particularly in generating critical medical documents such as medical histories, AI's performance needs thorough investigation.

This study hypothesizes that when provided with identical case materials, ChatGPT-4o can complete medical history documentation in less time while maintaining quality comparable to that of resident physicians. Through systematic comparison of documentation time and quality between the two, we aim to evaluate ChatGPT-4o's potential applications in actual clinical work and provide reference for AI's further development in healthcare.

The research findings may offer new insights into current medical documentation practices and provide novel solutions for optimizing resource allocation and improving work efficiency in healthcare institutions. Furthermore, this study will explore the limitations of AI applications in healthcare, providing direction for subsequent technological improvements and practical applications.

## Methods

### Study design

This study adopts a non-inferiority comparative design to evaluate the performance of ChatGPT-4o and residents in terms of medical record quality and efficiency. The study participants include five residents (3 males, 2 females) from the hematology department, a computer system equipped with ChatGPT-4o, and two attending physicians with more than 10 years of clinical experience, who will independently score the quality of medical records. Each resident and ChatGPT-4o will generate medical records based on the same case materials, and the attending physicians will score the quality of these records. The evaluation criteria include completeness, accuracy, logic, and professionalism, with clear and standardized scoring criteria to ensure consistency and objectivity in the assessment.

### Participants

- Residents: five residents currently undergoing standardized training in hematology, each with at least 1 year of clinical experience (average experience 2.4 ± 0.9 years, ensuring they possess sufficient skills in medical record collection and documentation. The residents' abilities in record-keeping will be pre-assessed to minimize individual differences that may influence the results. Selection criteria for residents included: (1) currently undergoing standardized training; (2) having at least 1 year of clinical experience; and (3) having recorded at least 30 hematology cases in the past 2 months.
- ChatGPT-4o: The latest version of ChatGPT-4o will be used to generate medical records. To ensure comparability, the system configuration and usage will be standardized, including the setting of prompts and generation parameters. Detailed configuration is provided in Appendix A. The main prompt template used was: “Based on the following transcribed doctor-patient dialogue, please generate a standard hematology medical history record, including chief complaint, present illness, past medical history, personal history, family history, physical examination, auxiliary examination, and diagnosis. Please ensure the content is complete, accurate, logically clear, and meets professional standards.”
- Attending Physicians: two experienced hematology attending physicians were responsible for scoring the medical records. Both had over 10 years of clinical experience and had been involved in resident training for the past 3 years. The scoring process was independent, with clear evaluation criteria to ensure consistency in the results.

### Data collection

- Interview Transcription: the resident will record the entire interview process while taking the patient's medical history, and the recorded content will be transcribed by specialized software (iFlytek Medical Version 1.2.0) into text, which will serve as the basis for the medical record. All transcriptions will undergo quality checks to ensure accuracy. The transcription process included: (1) audio collection (resident-patient dialogue); (2) automatic transcription (using speech recognition software); (3) manual correction (linguistic experts checking and correcting errors in automatic transcription); and (4) quality review (attending physicians confirming medical accuracy of the transcription). Transcription quality was assessed by comparison with the original audio, achieving an average accuracy rate of over 95%.
- Medical Record Documentation: each resident will independently document the medical record based on the transcribed text, and the same materials will be input into the ChatGPT-4o system to generate a medical record. The time taken for each resident and ChatGPT-4o to complete the medical record will be recorded to ensure comparability of time differences.
- Quality Scoring: the two attending physicians will independently score the medical records based on completeness, accuracy, logic, and professionalism. The scoring used a 100-point scale, and the final score will be the average of the two attending physicians' scores. Detailed scoring criteria are presented in Table 1 and Appendix B.

**Table 1:** Medical record quality scoring criteria.

**Scoring category**	**Scoring item**	**Scoring criteria**	**Maximum points**
General items	Chief complaint	Accurately extract main symptoms, concise and professional expression	6
	General requirements	Standardized format, complete content, clear structure	5
Core content	Present illness	Complete recording of onset time, triggers, clinical manifestations, medical visit process, treatment effects, etc.	30
	Past medical history	Accurate recording of all past diseases, surgeries, blood transfusions, allergies, etc.	10
	Personal history	Comprehensive recording of lifestyle habits, occupational exposure, social psychological factors, etc.	10
	Family history	Complete recording of family members' relevant disease history	5
Examination and diagnosis	Physical examination	Systematic and comprehensive physical findings, accurate description of abnormalities	20
	Auxiliary examination	Accurate recording of all examination results with important results highlighted	10
	Diagnosis	Diagnosis consistent with clinical manifestations, reasonable logical reasoning	4
**Total**			**100**

### Sample size calculation

The sample size calculation was based on a non-inferiority design. With an anticipated standard deviation of 10 points for quality scores, a non-inferiority margin (Δ) of 5 points (5% of the total score), a significance level (α) of 0.05, and a statistical power (β) of 0.80, we determined that each group required 63 cases. This 5-point margin was established through consultation with experienced attending physicians who considered a difference of < 5% in overall quality score to be clinically insignificant. To account for potential issues such as transcription quality, we included a final total of 65 cases to enhance the study's reliability. It is important to note that while only 5 residents participated, the unit of analysis was the medical record, not the number of participants, which aligns with the requirements of non-inferiority study design (13–15). We acknowledge the limitations of this sampling strategy and discuss them in detail in the discussion section.

### Evaluation indicators

- Medical Record Quality: scored by attending physicians, evaluating the completeness, accuracy, logic, and professionalism of the medical records.
- Documentation Time: the time taken by each resident and ChatGPT-4o to complete the medical record, measured in minutes.
- Medical Record Quality: scored by attending physicians, evaluating different aspects of the medical records across three main categories:

General Items (11 points): including chief complaint (6 points) and overall requirements (5 points)
Core Content (55 points): including present illness (30 points), past medical history (10 points), personal history (10 points), and family history (5 points)
Examination and Diagnosis (34 points): including physical examination (20 points), auxiliary examination (10 points), and diagnosis (4 points).

### Data preprocessing

To ensure that ChatGPT-4o could effectively process medical dialogues, we performed the following preprocessing on the transcribed text:

- Removal of filler words and repetitive content
- Standardization of medical terminology and abbreviations
- Organization of question-answer pairs in chronological order
- Addition of simple classification tags (such as “symptom description,” “treatment experience”) to unstructured dialogues

Preprocessing was conducted by a linguist with medical background and an information technology specialist, and reviewed by the project's supervising physician. These preprocessing steps ensured that the content input into ChatGPT-4o was structured clearly and contained the necessary medical information while preserving the original dialogue content as much as possible. The same preprocessed text was also provided to the residents as the basis for their history recording to ensure fair comparison.

### Data analysis

Data analysis was performed using SPSS 26.0 statistical software. First, paired *t*-tests was used to compare the time taken by residents and ChatGPT-4o to complete the records, assessing the statistical significance of any time differences. Wilcoxon signed-rank tests will be used to evaluate the quality differences between the two groups. Descriptive statistics will include means and standard deviations, and Kappa coefficients was used to analyze the consistency between the two attending physicians' scores to ensure the reliability and repeatability of the results. Additionally, in-depth analysis was conducted on items with significant differences, such as chief complaint and overall requirements, to identify specific aspects where ChatGPT-4o might need improvement.

### Ethical considerations

The study received IRB approval from Ruijin hospital's ethics committee (approval number: 2024-443). Written informed consent was obtained from all participants prior to their participation in this study, ensuring that participation is voluntary and that participants are fully informed. All patient information collected during the study was kept confidential and anonymized, used solely for research purposes.

## Results

### Comparison of medical record quality scores

Statistical analysis of the 65 cases was conducted to compare the performance of the resident group and the ChatGPT-4o group in each scoring category. The results showed in Table 2.

**Table 2:** Summary of comparative analysis across all evaluation metrics.

**Scoring category**	**Resident group Mean ±SD**	**ChatGPT-4o group Mean ±SD**	***p*-value**
Chief complaint	5.70 ± 0.27	5.50 ± 0.38	0.009^*^
Overall requirements	4.48 ± 0.33	4.31 ± 0.41	0.041^*^
Present illness	28.64 ± 1.14	28.42 ± 1.55	0.42
Past medical history	9.52 ± 0.54	9.65 ± 0.48	0.22
Personal history	9.42 ± 0.63	9.53 ± 0.57	0.26
Family history	4.83 ± 0.23	4.87 ± 0.20	0.49
Physical examination	19.25 ± 0.84	19.08 ± 0.93	0.27
Auxiliary examination	9.78 ± 0.26	9.81 ± 0.24	0.49
Diagnosis	3.75 ± 0.27	3.75 ± 0.29	0.97
**Total**	**89.57** **±2.66**	**88.94** **±3.13**	**0.25**

^*^Indicates *p* < 0.05, statistically significant difference. The Kappa coefficient between the two evaluators was 0.82.

**Figure 1:**
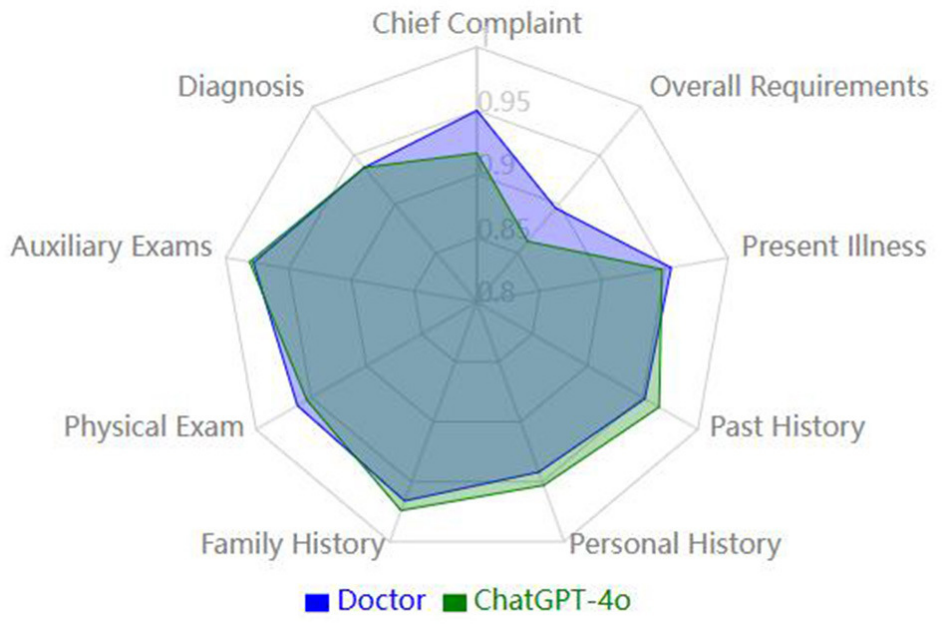
Comparison of medical history quality metrics (normalized).

Overall, the quality scores revealed that the resident and ChatGPT-4o groups performed similarly in several categories, with no significant differences between the groups. Specifically, no significant differences were found in the following categories: present illness, past medical history, personal history, family history, physical examination, auxiliary examination, and diagnosis (*p*-values: 0.42, 0.22, 0.26, 0.49, 0.27, 0.49, and 0.97, respectively) (Figure 1).

**Figure 2:**
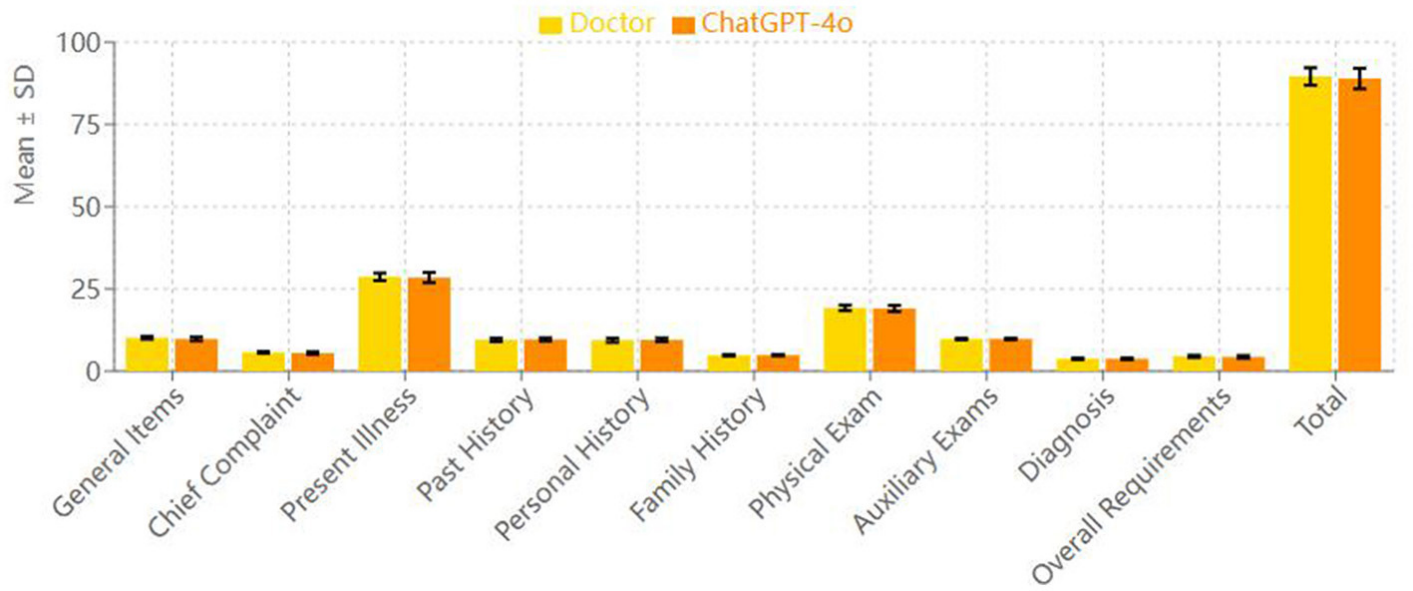
Comparison of mean scores (doctor vs. ChatGPT-4o).

However, in the “chief complaint” and “overall requirements” categories, the resident group scored significantly higher than the ChatGPT-4o group. In the “chief complaint” category, the resident group's mean score was 5.70 ± 0.27, while the ChatGPT-4o group's score was 5.50 ± 0.38, with a statistically significant difference (*p* = 0.009). In the “overall requirements” category, the resident group scored 4.48 ± 0.33 on average, while the ChatGPT-4o group scored 4.31 ± 0.41, which also showed a statistically significant difference (*p* = 0.041) (Figure 2).

In terms of total score across all categories, the resident group scored 89.57 ± 2.66, while the ChatGPT-4o group scored 88.94 ± 3.13. Paired *t*-test analysis showed no statistically significant difference between the two groups' total scores (*p* = 0.25), indicating that the overall quality of medical record documentation was comparable between the two groups.

### Non-inferiority comparison of medical record quality

To assess whether ChatGPT-4o's performance in medical record quality was not inferior to that of the resident group, a non-inferiority analysis was conducted. The non-inferiority margin (Δ) was set at 5 points, meaning a difference of < 5 points would indicate that ChatGPT-4o's performance was not inferior to the residents. The average total score for the resident group was 89.57, while the ChatGPT-4o group's average score was 88.94. The mean difference between the groups was 0.63 points, well below the non-inferiority margin (Δ = 5). The non-inferiority test results indicated that the quality score for ChatGPT-4o fell within the pre-established non-inferiority margin (*p* > 0.05), confirming that ChatGPT-4o's performance in medical record quality was not inferior to that of the residents.

### Comparison of medical record documentation time

The comparison of documentation time between the resident group and the ChatGPT-4o group showed that the resident group took an average of 893.2 seconds (~14.9 min) to complete the medical records, with a standard deviation of 28.0 s. In contrast, the ChatGPT-4o group completed the medical records in an average of 40.1 ± 4.4 s. Paired *t*-test analysis revealed that the time difference between the two groups was statistically significant (*p* < 0.001), indicating that ChatGPT-4o demonstrated significantly better efficiency in medical record documentation compared to the resident group.

## Discussion

This study aims to assess the performance of ChatGPT-4o and resident physicians in terms of medical record efficiency and quality (16). The results indicate that while ChatGPT-4o maintains a comparable quality of medical records to the residents, it significantly reduces the time required for documentation. Specifically, ChatGPT-4o required only 40 s on average, whereas the resident physicians took ~15 min. This difference was statistically significant, highlighting ChatGPT-4o's clear advantage in time efficiency. However, it is important to note that the time required to process dialogue and correct transcription errors from speech recognition before generating the final record should also be considered. Improved speech recognition technology will be crucial for directly transcribing consultation processes into medical records through AI systems.

Although ChatGPT-4o demonstrated remarkable time efficiency, its quality scores were comparable to those of the residents. No significant differences were observed between the two groups in present illness, past medical history, personal history, family history, physical examination, auxiliary examinations, and diagnosis. However, in the “chief complaint” and “overall requirements” categories, the resident group scored significantly higher than the ChatGPT-4o group (*p* = 0.009 and *p* = 0.041, respectively). This suggests that, in these specific dimensions of medical record documentation, the residents performed better. These areas are more dependent on language proficiency and writing skills, and it is expected that AI models, including ChatGPT, may face some challenges in language generation, especially in non-native languages like Chinese.

From the perspective of non-inferiority analysis, although the residents scored slightly higher on certain items, ChatGPT-4o did not perform worse overall in terms of medical record quality. There was no statistically significant difference in total scores (*p* = 0.25), and the average difference between the groups was much smaller than the pre-set non-inferiority margin (Δ = 5 points). This suggests that ChatGPT-4o can achieve a level of record quality similar to that of the resident physicians.

This finding holds significant clinical implications in the context of healthcare settings with heavy physician workloads (17). The high efficiency of ChatGPT-4o in record-keeping means it can alleviate physicians' burden while maintaining the quality of medical records, offering considerable potential to improve the overall efficiency of the healthcare system. ChatGPT-4o could be widely applied in various clinical settings, especially in time-sensitive environments like emergency departments and intensive care units, where quick and efficient record support is critical. Additionally, in primary care settings, particularly in areas lacking experienced physicians, ChatGPT-4o could assist junior doctors in completing high-quality medical records, thus improving the quality of medical services.

However, despite the excellent performance of ChatGPT-4o, its clinical application faces several ethical challenges (18–20). Medical records involve sensitive patient information, and ensuring data security and privacy protection is a critical concern. Furthermore, over-reliance on AI could potentially diminish physicians' clinical reasoning abilities, thus impacting overall medical decision-making. Therefore, a balance must be struck between the use of technology and physician involvement to ensure clinical judgment is not compromised. Moreover, ethical review in medical record-keeping should ensure patient informed consent and clearly define the scope of data usage. Additionally, maintaining the model's focus and consistency remains a challenge in practical applications.

The limitations of this study include a small sample size, the focus on the hematology field, and the inability of the study design to cover all potential clinical complexities (21). In terms of sample selection, this study involved only five residents from a single specialty (hematology), which may limit the generalizability of the results. Future research should expand the sample size and explore the performance of ChatGPT-4o in other specialties. Each resident's background and experience level may influence their recording capabilities, and despite our attempt to minimize these differences through pre-assessment, selection bias may still exist. Additionally, there may be subjectivity in the standardization and scoring process, and while we attempted to reduce this through clear scoring criteria and independent scoring by two evaluators, the subjectivity of scoring remains inevitable. All clinicians in this study were from Ruijin Hospital, which may also limit the geographical representativeness of the results. Moreover, it is important to evaluate ChatGPT-4o's ability to handle complex cases and rare conditions, which would help comprehensively assess its applicability in clinical practice.

One promising research direction could involve integrating ChatGPT-4o with other AI systems, such as image recognition and retrieval-augmented generation (RAG) technologies, to create a multimodal clinical decision support system. This system could not only optimize medical record documentation but also provide real-time diagnostic suggestions and treatment plans. Such an integrated system would be particularly effective in assisting physicians with decision-making, especially in complex or rare cases.

## Conclusion

This study provides strong evidence for the application of AI in medical history documentation, demonstrating the potential of ChatGPT-4o to improve clinical efficiency while maintaining medical history quality. As technology continues to develop, ChatGPT-4o or similar AI systems are expected to play a broader role in the healthcare field. However, how to maintain medical ethics and doctors' clinical abilities while applying these technologies will remain an ongoing and important issue.

## Data Availability

The original contributions presented in the study are included in the article/Supplementary material, further inquiries can be directed to the corresponding authors.
