# Supplementary material for: Comparison of medical history documentation efficiency and quality based on GPT-4o: a study on the comparison between residents and artificial intelligence
Source: Front Med (Lausanne). 2025 May 14;12:1545730. doi: 10.3389/fmed.2025.1545730 (PMC12116629; doi:10.3389/fmed.2025.1545730)
Supplement: Supplementary file 1 [file Data_Sheet_1.docx]

**Appendix A: ChatGPT-4o Configuration and Prompt Settings**

In this study, we used the following configuration settings to run ChatGPT-4o:

1. **Model Used**: GPT-4o (June 2024 version)
2. **Temperature**: 0.2 (to ensure consistency of outputs)
3. **Maximum Output Length**: 4000 tokens
4. **Generation Strategy**: Probability-based selection (Nucleus Sampling with p=0.8)

The standard prompt template was as follows:

Based on the following transcribed doctor-patient dialogue, please create a standardized hematology medical history record. Please ensure that all of the following sections are included:

1. Chief Complaint

2. Present Illness

3. Past Medical History

4. Personal History

5. Family History

6. Physical Examination

7. Auxiliary Examination

8. Diagnosis

Please ensure the content is complete, accurate, logically clear, and meets professional standards. The record should follow standard format requirements for medical institutions in China.

Transcribed doctor-patient dialogue:

[Transcribed text inserted here]

For each case, we ensured that the prompt template remained consistent, with only the transcribed text changing, to ensure consistency in evaluation.

# Appendix B: Detailed Scoring Criteria

## 1. General Items (11 points total)

### 1.1 Chief Complaint (6 points)

- **6 points**: Completely accurate extraction of main symptoms, presented with concise and professional expression. Includes exact duration and precise description of primary symptoms.
- **5 points**: Accurate extraction of main symptoms, but expression slightly verbose or uses less precise medical terminology.
- **4 points**: Basic extraction of main symptoms, but with minor omissions or imprecise time course.
- **3 points**: Symptom description not accurate enough, with obvious omissions or includes irrelevant information.
- **2 points**: Severely inadequate symptom description, major symptoms missing or incorrectly characterized.
- **1 point**: Incorrect chief complaint content or inconsistent with patient's condition.
- **0 points**: No chief complaint provided.

### 1.2 Overall Requirements (5 points)

- **5 points**: Perfect formatting, complete content organization, clear structure with logical progression, and proper use of medical terminology.
- **4 points**: Good formatting with minor inconsistencies, content well-organized with appropriate structure.
- **3 points**: Acceptable formatting but some organizational issues, adequate structure with some logical flaws.
- **2 points**: Poor formatting with significant organizational problems, unclear structure.
- **1 point**: Major formatting and organizational deficiencies, structure difficult to follow.
- **0 points**: No discernible organization or structure.

## 2. Core Content (55 points total)

### 2.1 Present Illness (30 points)

- **28-30 points**: Exemplary documentation including:
  - Precise onset time with exact date
  - Clear identification of triggering factors
  - Comprehensive description of symptom evolution
  - Complete timeline of medical consultations
  - Detailed treatment history with specifics on medications, dosages, and effects
  - Clear description of symptom progression or resolution
  - Logical narrative flow
- **25-27 points**: Very good documentation with minor omissions in one area
- **22-24 points**: Good documentation with minor omissions in two areas or moderate omission in one area
- **19-21 points**: Satisfactory documentation with several minor omissions
- **16-18 points**: Basic documentation with significant details missing
- **13-15 points**: Poor documentation with major elements missing
- **10-12 points**: Very poor documentation with only minimal information provided
- **<10 points**: Severely deficient, missing critical information

### 2.2 Past Medical History (10 points)

- **10 points**: Complete history including all:
  - Previous diseases with dates of diagnosis and treatment
  - All surgeries with dates
  - Blood transfusion history
  - Complete allergy history (drug, food, environmental)
  - Immunization history when relevant
  - Previous hospitalizations
- **8-9 points**: Nearly complete with minor omissions
- **6-7 points**: Good coverage of major conditions but missing some details
- **4-5 points**: Major conditions mentioned but lacking significant details
- **2-3 points**: Several major elements missing
- **0-1 points**: Severely incomplete or missing

### 2.3 Personal History (10 points)

- **10 points**: Comprehensive personal history including:
  - Detailed lifestyle habits (smoking, alcohol, diet, exercise)
  - Complete occupational exposure history
  - Thorough social history (living conditions, socioeconomic factors)
  - Psychological factors when relevant
  - Travel history when relevant to presenting complaint
- **8-9 points**: Nearly complete with minor omissions
- **6-7 points**: Good coverage but missing some details
- **4-5 points**: Basic information provided but lacking significant details
- **2-3 points**: Several major elements missing
- **0-1 points**: Severely incomplete or missing

### 2.4 Family History (5 points)

- **5 points**: Complete family history including:
  - Three-generation pedigree when relevant
  - Specific diseases in family members with relation to patient
  - Ages of onset for relevant conditions
  - Current health status or age at death of immediate family members
- **4 points**: Good family history with minor omissions
- **3 points**: Basic family history covering immediate family
- **2 points**: Incomplete family history with significant omissions
- **1 point**: Severely limited family history
- **0 points**: Missing family history

## 3. Examination and Diagnosis (34 points total)

### 3.1 Physical Examination (20 points)

- **18-20 points**: Comprehensive physical examination including:
  - Complete vital signs (temperature, pulse, respiration, blood pressure)
  - Systematic examination of all body systems
  - Detailed description of all abnormal findings
  - Precise terminology for all physical findings
  - Quantitative measurements where appropriate
- **15-17 points**: Nearly complete examination with minor omissions
- **12-14 points**: Good examination but missing some details
- **9-11 points**: Basic examination documenting major systems
- **6-8 points**: Incomplete examination with significant omissions
- **3-5 points**: Very limited examination
- **0-2 points**: Severely deficient or missing

### 3.2 Auxiliary Examination (10 points)

- **9-10 points**: Complete documentation of all auxiliary examinations including:
  - All laboratory results with reference ranges
  - All imaging studies with key findings highlighted
  - Specialist consultations with findings
  - Proper categorization and organization of results
  - Emphasis on abnormal findings
- **7-8 points**: Nearly complete with minor omissions
- **5-6 points**: Good coverage of major results but missing some details
- **3-4 points**: Basic results provided but lacking organization or emphasis
- **1-2 points**: Severely incomplete auxiliary examination documentation
- **0 points**: Missing auxiliary examination documentation

### 3.3 Diagnosis (4 points)

- **4 points**: Excellent diagnosis including:
  - Primary diagnosis with appropriate specificity
  - All relevant secondary diagnoses
  - Clear differential diagnosis when appropriate
  - Evidence-based reasoning connecting symptoms to diagnoses
- **3 points**: Good diagnosis with appropriate primary and most secondary diagnoses
- **2 points**: Adequate primary diagnosis but incomplete secondary diagnoses
- **1 point**: Incomplete or partially incorrect diagnosis
- **0 points**: Missing or completely incorrect diagnosis

## Case Scoring Examples

To illustrate the practical application of these scoring criteria, below are two anonymized case scoring examples:

### Case Example 1: Patient with Lymphoma (ID: A023)

| **Scoring Category** | **Evaluator A Score** | **Evaluator B Score** | **Average Score** | **Scoring Rationale** |
| --- | --- | --- | --- | --- |
| Chief Complaint | 5 | 6 | 5.5 | Accurate symptom description but slightly verbose |
| Overall Requirements | 4 | 5 | 4.5 | Well-organized with minor formatting inconsistencies |
| Present Illness | 29 | 28 | 28.5 | Clear timeline, comprehensive symptom description, but treatment effect description slightly insufficient |
| Past Medical History | 10 | 9 | 9.5 | Comprehensive and detailed, but allergy history could be more detailed |
| Personal History | 9 | 10 | 9.5 | Excellent detail on lifestyle and occupational factors |
| Family History | 5 | 5 | 5.0 | Complete three-generation coverage with specific conditions |
| Physical Examination | 18 | 19 | 18.5 | Comprehensive with detailed lymph node examination |
| Auxiliary Examination | 10 | 9 | 9.5 | Complete laboratory and imaging results with excellent organization |
| Diagnosis | 4 | 3 | 3.5 | Clear primary diagnosis with good differentials |
| **Total** | **94** | **94** | **94.0** | **Excellent quality, comprehensive and well-structured** |

### Case Example 2: Patient with Leukemia (ID: B047)

| **Scoring Category** | **Evaluator A Score** | **Evaluator B Score** | **Average Score** | **Scoring Rationale** |
| --- | --- | --- | --- | --- |
| Chief Complaint | 5 | 4 | 4.5 | Accurate but included some unnecessary details |
| Overall Requirements | 4 | 4 | 4.0 | Good organization but some inconsistent formatting |
| Present Illness | 26 | 25 | 25.5 | Good symptom progression but timeline slightly unclear |
| Past Medical History | 8 | 9 | 8.5 | Good detail but missing some medication history |
| Personal History | 7 | 8 | 7.5 | Adequate coverage but limited occupational exposure details |
| Family History | 4 | 4 | 4.0 | Good coverage of immediate family, limited extended family |
| Physical Examination | 17 | 16 | 16.5 | Good systematic examination with minor omissions |
| Auxiliary Examination | 9 | 8 | 8.5 | Good laboratory results but could highlight abnormals better |
| Diagnosis | 3 | 3 | 3.0 | Correct primary diagnosis with limited secondary diagnoses |
| **Total** | **83** | **81** | **82.0** | **Good quality with room for improvement in timeline clarity and occupational details** |

These examples demonstrate how the scoring criteria are applied in practice. The cases show different levels of performance across categories and illustrate the consistency between evaluators despite minor scoring variations. The detailed rationale provides insight into specific strengths and weaknesses of each medical record.
